# Are Movement Artifacts in Magnetic Resonance Imaging a Real Problem?—A Narrative Review

**DOI:** 10.3389/fneur.2017.00232

**Published:** 2017-05-30

**Authors:** Inger Havsteen, Anders Ohlhues, Kristoffer H. Madsen, Janus Damm Nybing, Hanne Christensen, Anders Christensen

**Affiliations:** ^1^Department of Radiology, Copenhagen University Hospital Bispebjerg, Copenhagen, Denmark; ^2^Department of Clinical Engineering Diagnostic Imaging Section, Copenhagen University Hospital Rigshospitalet, Copenhagen, Denmark; ^3^Danish Research Centre for Magnetic Resonance, Copenhagen University Hospital Hvidovre, Hvidovre, Denmark; ^4^Department of Neurology, Copenhagen University Hospital Bispebjerg, Copenhagen, Denmark

**Keywords:** acute stroke imaging, dynamic magnetic resonance imaging, motion artifacts, noise reduction, motion tracking

## Abstract

Movement artifacts compromise image quality and may interfere with interpretation, especially in magnetic resonance imaging (MRI) applications with low signal-to-noise ratio such as functional MRI or diffusion tensor imaging, and when imaging small lesions. High image resolution has high sensitivity to motion artifacts and often prolongs scan time that again aggravates movement artifacts. During the scan fast imaging techniques and sequences, optimal receiver coils, careful patient positioning, and instruction may minimize movement artifacts. Physiological noise sources are motion from respiration, flow and pulse coupled to cardiac cycles, from the swallowing reflex and small spontaneous head movements. Par example, in resting-state functional MRI spontaneous neuronal activity adds 1–2% of signal change, even under optimal conditions signal contributions from physiological noise remain a considerable fraction hereof. Movement tracking during imaging may allow for prospective correction or postprocessing steps separating signal and noise.

## Background

Movement artifacts are an inherent problem to magnetic resonance imaging (MRI) technology where low signal and sensitivity to motion are obstacles driving the development of ever faster sequences, e.g., gradient echo, and finer detection equipment, e.g., multichannel phased array coils, since the very beginning of nuclear magnetic resonance (NMR) imaging (1). A brief history of medical imaging may be found in Ref. (2).

### Why Is Movement a Problem?

Movement artifacts in MRI degrade image quality and may lead to misinterpretation especially in MRI acquisitions with low signal-to-noise ratios (SNRs), or for small lesion pathology. In MRI sequences with robust visual interpretation, simple motion artifacts can be identified as, e.g., ghosting or blurring. In dynamic MRI scans, motion artifacts can cause signal changes that may severely confound statistical analysis rendering results unreliable (3, 4).

### Movement Artifacts Interfere with Image Interpretation in Dynamic and Static MRI

#### Functional MRI (fMRI)

**Figure 1:**
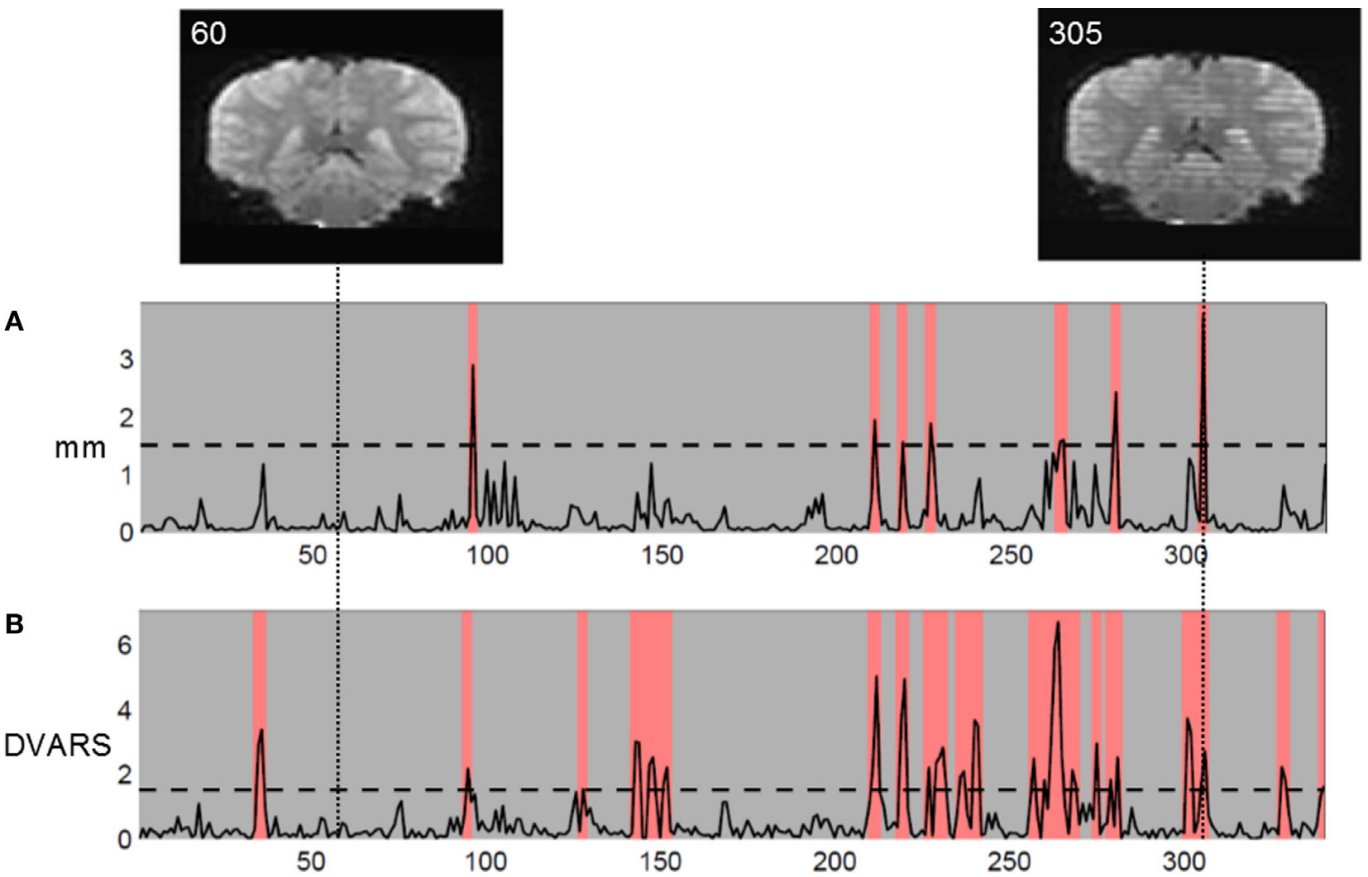
**Coronal reconstruction of echo planar images (EPI) in volume without motion (volume 60, left) and volume with motion artifacts (volume 305, right)**. The striped appearance of volume 305 arises from the interleaved EPI sequence used. Two movement measures are shown: **(A)** Euclidian translational displacement in millimeter and **(B)** DVARS (percent mean signal change) as defined in Ref. (5). In this study of children, with liberal chosen movement thresholds, we discarded volumes exceeding threshold and marked in red.

Functional MRI measures subtle changes in local blood oxygenation and flow related to neural activity. Head motion artifacts can cause signal changes, Figure 1. In the worst case, motion-related signal changes may be correlated with activation of interest in task-based fMRI rendering results difficult to interpret or in resting-state where motion-related signal changes may be confused with correlations between regions when measuring functional connectivity (3–6). By carefully mapping isolated head movement artifacts, spatial patterns resembling the default mode network were found (3). Also movement induced signal changes introduce spatiotemporal structured noise that invalidate the typical assumptions of independent and identically distributed Gaussian noise in the statistical analysis (7).

#### Diffusion-Weighted Imaging (DWI)

**Figure 2:**
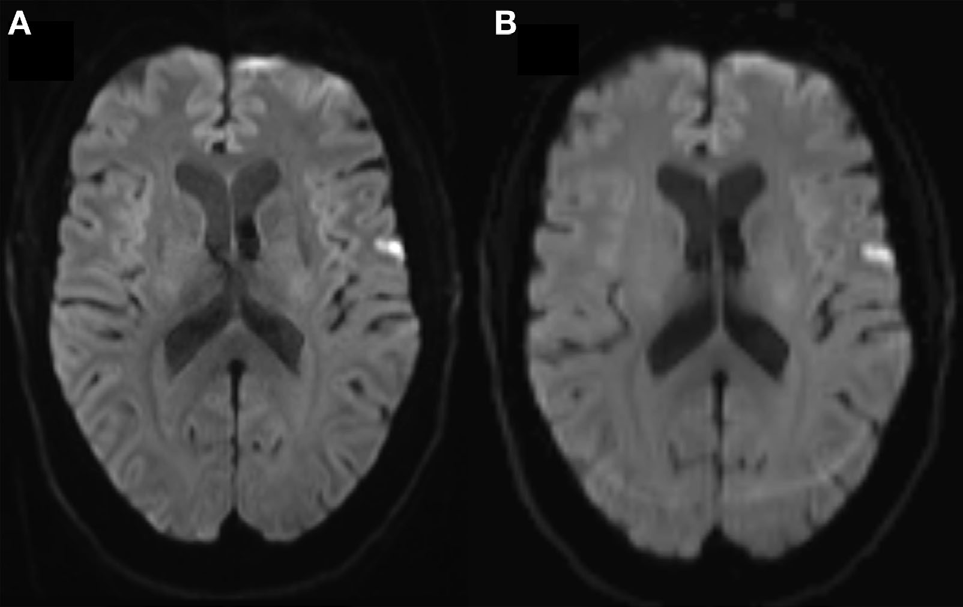
**(A)** Three and **(B)** 20-gradient direction diffusion-weighted imaging (DWI) images of 68 years female with 2 h lasting transient ischemic attack symptoms including right hand paresis and slurred speech. In **(B)**, note the occipital ring artifact, blurred contours of the right-sided cortical diffusion lesion and blurred cortex outline due to motion. Three-gradient direction DWI had acquisition time 2 min and 7 s and 20-gradient direction DWI had 4 min and 39 s, both were standard vendor protocols.

Diffusion-weighted imaging shows directional variation of diffusion restriction in diffusion-weighted images. Movement artifacts cause misalignment of data and introduce noise in the images rendering results unreliable (8). The main compromise usually stands between resolution and acquisition time. DWI has long acquisition times with repetition times up to 10 s which increases sensitivity to motion artifacts. For detection of focal diffusion restriction, one usually uses three orthogonal diffusion directions only, with usual 1–2 mm axial resolution and acquisition times around 2 min. Diffusion tensor imaging (DTI) used for, e.g., fiber tracking and determination of white mater integrity needs imaging along at least six gradient directions, usually 20–60. DTI has longer acquisition times, usually 4–5 min, rendering it more susceptible to motion artifacts than 3-gradient direction DWI, Figure 2. In DWI, artifacts due to physiological noise are usually minor and can be handled by gating (9–11).

#### Arterial Spin Labeling (ASL)

Arterial spin labeling is a perfusion imaging technique that uses endogenous blood water labeled as “paramagnetic tracer” to estimate cerebral blood flow. One labels blood water prior to inflow into the imaging region and subtracts labeled images from control images to find a measure proportional with cerebral blood flow. Bulk motion during free breathing introduces additional blurring. Breath-hold timing and background suppression schemes enhance image quality using series of additional saturation and inversion pulses (12).

#### Structural Images

In structural images movement, artifacts are a smaller problem, here the strong SNR enables visual acuity to robustly differentiate between anatomical structure and artifact. Yet, challenges remain especially in areas with high intrinsic motion, e.g., cardiac MRI encounters both cardiac pumping and respiration. Head motion has been shown to compromise T1-derived volumetric measurements of cortical thickness, where a seeming reduction imitates cortical atrophy (13).

#### Small Lesions

Small lesions, 3 mm or below, are challenging to image with high fidelity and to confidently categorize (14, 15), yet evidence grows of their clinical importance as, e.g., small stroke suspect lesion presence is associated with increased mortality and morbidity (16). Intra- and interrater agreement show deviations <5% and 2 ml for acute ischemic DWI lesions over 15 ml (17). For smaller lesions in a combined minor stroke and TIA cohort with mean DWI lesion volume 3.4 ml, measurement intra- and interrater agreement were very good (ICC 0.96 and 0.94) (18). Still, 3.4 ml corresponds to 1.5 cm lesion length using simple cubic calculation. While these results are excellent, there still is a way to lesions around 3 mm, the usual cutoff value for ischemic lesion inclusion (19), these would have volumes of 0.027 ml. Only recently, the Standards for Reporting Vascular Changes on Neuroimaging group has included lesions below 3 mm as potential signs of small vessel disease (14). The goal is to image structures with enough detail and minimal distortion to achieve proper identification. One path is noise reduction.

### Higher Field Strengths Yield Higher Resolution and Higher Sensitivity to Motion Artifacts

There is a general trend toward higher magnetic field strengths (1). At 7 T, angiographic MRI studies using susceptibility-weighted imaging (20) or Time Of Flight (21) achieve resolutions similar to CT (about 0.4 mm in-plane) without contrast agents or ionizing radiation. The increase in imaging sensitivity at higher field strengths comes with an increase in sensitivity to physiological noise and motion, i.e., the proportion of noise increases even without increasing resolution. Higher B fields allow higher resolution, where even smaller movement artifacts compromise image quality. Also high resolution demands long acquisition times and motion artifacts worsen with longer acquisition times. The problem remains in future.

### Aim

This text aims to assess if subject-related movement artifacts in MRI are problematic, i.e., interfere with interpretation, to identify where this is the case, investigate the magnitude of movement artifacts compared with MR signal and other noise sources, and to explore strategies to attenuate or circumvent movement artifacts.

This review is rooted in neuroimaging in a clinical context but extends into the realm of research, as many clinicians are involved in research budding from clinics striving to improve current practice. The intended audience is the interested neurologist in the interface between clinics and neuroscientific research. The review’s scope is introductory, to provide a background for understanding the underlying causes of motion artifacts and strategies for their mitigation. This is a narrative review based on a practical approach; a systematic literature review is beyond the scope of this text.

## The Signal and the Noise

### Outlining the Problem: Accurate Imaging at High Resolution

Small objects are most susceptible to motion artifacts. If the resolution, voxel size, is near or larger than the imaged objects, their contours appear smeared or blurred and the effect is called partial volume. Similarly, movement artifacts degrade image quality, because some voxels will be moved to another part of the object that may have different signal intensity, e.g., in the brain a white matter voxel is moved into gray matter or cerebrospinal fluid filled cavities as the lateral ventricles. Thus, motion artifacts are most prominent at contrast edges (22), i.e., the border between the brain and the skull or air-filled sinuses, borders between gray and white matter and around the lateral ventricles.

In image acquisition, in-plane acceleration schemes (23–25) are attractive as they allow decreasing the length of readout trains. This greatly reduces distortions allowing shorter echo times and higher resolutions to be achieved. However, in this context, it is important to note that these acceleration schemes come at the price of reduced SNR (25–27) and importantly can lead to increased motion sensitivity, in particular if motion occurs during the reference/auto-calibrating signal scans. Furthermore, accelerated imaging may cause complicated motion artifacts that are more difficult to identify. Simultaneous multislice (SMS) acquisition schemes (28, 29) allow speeding up echo planar imaging (EPI) acquisition with little or no penalty in the SNR for moderate acceleration factors. When compared to in-place acceleration schemes, SMS is typically considered to increase motion sensitivity to a lesser extent; however, it should be noted that SMS can also complicate the identification of motion artifacts as they will affect several slices simultaneously and may lead to reconstruction artifacts.

One may attempt to correct for motion artifacts, i.e., motion-induced voxel misplacement relative to adjacent structures, by retrograde realignment of the acquired image slices. The procedure is called six parameter rigid body transformation and mitigates motion-related noise (30, 31). One assumes that the imaged volume, i.e., the head, is a rigid body and that its movements can be described with six vectors, three translational along orthogonal axes, and three rotational. Retrograde calculations of translational and rotational movements are based on the assumption that motion happens only between volumes. In reality, most sequences use most of the time for either magnetization or readout. Also the method does not account for non-linear motion effects or movements in previous scans and their effects on field inhomogeneity and spin history (32). Motion artifacts, here mostly head motion, cause local changes in the magnetic field showing for echo planar sequences as warping in the phase encoding direction and for spiral acquisition as blur (22). Yet, the six-parameter rigid body transformation greatly reduces motion effects and is a common preprocessing step in fMRI.

### Scaling the Signal and the Noise

#### Back to Basics—How Are MR Images Made?

When an object is within a strong magnetic field, one may send in a radio wave and receive an echo (the MR signal). The echo is determined by two physical constants, T1 and T2. Each tissue has unique T1 and T2 relaxation curves.

The MRI signal is created by a strong static magnetic field (B_0_) formed by a superconductive coil combined with one or more radiofrequency (RF) fields (B_1_) created through the application of RF pulses and several weak magnetic fields generated by gradient coils.

For anatomic MR signal (echo) localization in voxels, one forms a grid with three orthogonal directions: the RF excitation pulse creates a weaker B_1_ field. A slice-selective gradient cuts the B_1_ field into two-dimensional (2D) slices, and in each 2D slice magnetic field gradients encode phase and frequency forming cubes (voxels) with unique anatomic localization. The RF pulse is tuned to the NMR frequency of hydrogen, which is determined by the strength of the B_0_ field and the gyromagnetic ratio of hydrogen. When a patient enters the scanner, the magnetic moments of protons in the body tend to align with the B_0_ field. The RF pulse is applied and forces the magnetic moments to precess at their resonant frequency creating the B1 field. This precession and its decay after the RF pulse is switched off (the echo) are detected by one or more receive coils as the signal. For more details, see, e.g., Ref. (2, 33).

Hydrogen (1H) is the most abundant and commonly used. 19F, 31P, 7L1, 129Xe, 23Na, 13C, and 17O are examples of other nuclei that possess the required spin property, and each requires its own RF pulse tuned to its frequency.

#### How Does the MR Signal Relate to Field Strength?

Higher magnetic field strengths improve image SNR and contrast-to-noise ratio (CNR) yielding higher resolution. Images with higher resolution are more sensitive to motion artifacts. Table 1 shows that higher spatial resolution is achievable at higher field strength and that the spatial resolution of fMRI is inferior to the anatomical resolution because signal changes are small, only a few percent of the available signal (Table 2). All other conditions equal, higher spatial resolution requires longer acquisition times. Presumably, one could obtain higher resolution in the same or shorter time if one increases the SNR/CNR accordingly.

**Table 1:** **Spatial resolution**.

B (T)	Resolution (mm^3^)
3 T anatomical images	1 × 1 × 1
3 T functional MRI (fMRI) and perfusion contrast	2 × 2 × 2^a^
7 T anatomical images	0.5 × 0.5 × 0.5
7 T fMRI	1 × 1 × 1^a^

*From Ref. (1)*.

*^a^Spatial resolution is inferior to anatomical because the small signal changes, only a few percent of the available signal. The use of higher resolutions generally reduces both image signal-to-noise ratio and contrast-to-noise ratio*.

**Table 2:** **Contributions to rs-fMRI signal change in whole brain gray matter at voxel level in 7 T**.

Low frequency drift due to scanner instability	3.2%
Thermal noise	2.3%
Spontaneous neuronal activity	1.9%
RETROICOR^a^	0.1%
Cardiac rate	0.1%
Respiration volume per unit time	0.1%

*From Ref. (34)*.

*^a^Eight regressors correlated with physiological activity*.

### fMRI Signal

The anatomical basis of the fMRI signal are perfusion and oxygenation-related local changes in venous blood [blood oxygenation level-dependent (BOLD) signal] in the cortex and pial vessels related to local neuronal activity (35, 36). Deoxyhemoglobin is paramagnetic and oxyhemoglobin is diamagnetic. The paramagnetic deoxyhemoglobin causes a focal artifact of signal loss in T2*-weighted sequences because it causes a focal inhomogeneity in the magnetic field that increases T2* decay. The metabolic demand of neural activity increases local perfusion and oxygenation, decreasing local deoxyhemoglobin concentration. The relative absence of deoxyhemoglobin and its related signal loss is seen as BOLD signal increase. For more details, see, e.g., Ref. (37). Movement may veil or obliterate these subtle local field homogeneity changes.

The changes in BOLD signal amplitude are only a few percent of the signal and are too small for visual assessment. They require statistical analysis for detection (Table 2). fMRI precision estimation depends not only on image SNR but also on the signal stability on repetition of the image acquisition as reflected in the temporal SNR (tSNR) (38).

### Breaking Down the Noise into Its Components—Nuisance Modeling

In fMRI signal, variability may stem from four principal sources as thermal and scanner noise arising from system instabilities, physiological noise of BOLD origin (spontaneous neural activity), and other physiological noise arising from subject motion, cardiac cycles, and respiration (34).

Table 2 shows noise sources’ relative contribution to resting-state fMRI signal changes.

Thermal noise is related to the scanning process and has white noise characteristics (uniform power spectral density) and originates from both the brain tissue and from detector electronics. It can be reduced using high B-fields and multichannel detector array receive coils.

Non-thermal, physiological noise sources generally cause signal fluctuations that scale with the absolute signal strength (39–41). In fMRI, the noise sources, physiological and non-physiological, need to be properly characterized and separated from the signal (34, 42). Otherwise they limit the improvements in detection sensitivity available with high B-fields (27, 43).

#### Movement-Related Noise and Its Size

Even healthy and cooperative adults show spontaneous head movements up to a millimeter (32). Friston and colleagues (32) divided movement-related signal components into differences in the position of the object in the scanner and differences due to the history of the position of the object. Table 3 shows displacement sizes for respiratory, cardiac, and head motion.

**Table 3:** **Displacement sizes**.

Respiratory motion of the diaphragm	Several cm
Respiratory motion of the chest wall	Several mm
Cardiac motion	>1 cm (44)
Head motion	1 mm (32)
Brain pulsation	0.1 mm (45)

#### Respiration and Cardiac Cycles

During normal *breathing*, the diaphragm moves several centimeters and the chest wall several millimeters. Most imaging strategies involve tracking of the respiratory cyclic motion and imaging within a chosen interval of the cycle (gating).

*Cardiac motion and arterial pulsation* have implications for imaging, especially for heart and brain studies. Cardiac pumping consists of longitudinal and radial contraction and causes displacements measuring over 1 cm in healthy individuals (44). Also beat-to-beat variations in blood flow may cause artifacts (46). Further reading on motion in cardiovascular imaging can be found in Ref. (47).

Brain pulsation cause non-rigid displacements of up to 0.1 mm in some brain regions (45). Timing the MRI pulse with respiratory and cardiac cycles (gating) may be necessary when imaging at submillimeter resolutions in research settings.

### Other fMRI- and DWI-Relevant Artifacts

#### Susceptibility Artifacts at Tissue Boundaries

The EPI sequence used for fMRI and DWI is vulnerable to susceptibility artifacts. Differences in tissue magnetic susceptibility cause field inhomogeneity at tissue boundaries, which cause spins to dephase faster and frequency shifts that produce low signal areas. Bone and air have much lower magnetic susceptibility than most soft tissues; thus, the signal loss is most pronounced at brain–air or brain–bone interfaces.

## Handling the Noise

To achieve as good and reliable data as possible to draw valid conclusions from it is an advantage to know if and when motion has occurred and its extent. Ideally, external motion tracking is preferable to motion estimation from data itself as motion may compromise the acquired data. The effective tracking system aims to provide real-time tracking with subpixel accuracy and must not introduce extra artifacts (48).

### General Strategies to Avoid or Reduce Physiologic Noise—Quick and Snug

The use of fast imaging sequences and optimal receive coils minimizes acquisition times and hence subject motion. One may consider using shorter protocols with for restless patient groups. Usually these protocols have a compromise between resolution and acquisition time, they are useful in, e.g., acute settings where quick information without detail is better than no information. Careful considerations on comfortable positioning of patients in the scanner, instruction and reminding of the importance of staying still during the scan are essential. Sedation or anesthesia may be necessary for difficult cases. Table 4 summarizes common sources of motion artifacts and practical tips.

**Table 4:** **Common sources of motion artifacts and practical tips**.

Motion source	Mitigation strategy
Situational subject motion	Protocol design matches population (e.g., shorter protocols in acute settings)
	Patient preparation including management of pain, claustrophobia, or other discomfort
	Information, scanner familiarization
	Comfortable positioning and optimal head support by padding
	Reminders
	Structural magnetic resonance imaging: sedation, if clinically indicated
	Functional MRI: task pretraining
Physiological	Monitoring
	Imaging in chosen intervals on respiratory/cardiac function curve
	Skip data with motion above predefined threshold
	*Post hoc* motion correction as estimated from data

#### Shielding

Motion artifacts occur in the phase direction. Saturation bands are areas where RF pulses are used to suppress MR signal from moving tissues outside the structure one wants to image, e.g., if on axial spine images the phase direction is anterior–posterior, the saturation band is placed to cover the throat and esophagus to avoid motion artifacts from swallowing on the spine.

#### Alternative Acquisition Patterns in k-Space

Motion artifacts on sequences with simple linear data acquisition in k-space result in concentrated motion artifacts in certain areas of the scan according to the time of the motion event, e.g., a single slice becomes unreadable. Alternative, e.g., propeller-shaped data acquisition patterns fill the center of the k-space repeatedly and thereby enabling motion correction between the propeller blades if inconsistencies occur (49, 50).

### Handling Noise from Respiration and Cardiac Cycles in Advanced Neuroimaging

In functional MRI, changes in respiration rate and depth over time cause non-neuronal BOLD signal changes, i.e., the varying TR due to, e.g., breath hold will cause T1 effects in BOLD imaging that are difficult to handle. The most common approach in neuroimaging is a combination of careful instruction, respiration monitoring, skip-and-redo, and *post hoc* modeling to eliminate respiration-induced signal changes (51). Other specific strategies to handle artifacts from respiration are respiratory gating or triggering, respiratory compensation or phase re-ordering (52) and navigator echoes (53).

Cardiac cycles may be tracked either centrally with ECG nodes or peripherally with pulse oximeters. While traveling in the arteries, the pulse cycle is delayed and deformed with distance to the heart, so the tracking position depends on what one wants to image: imaging the heart will yield the best results by tracking the cardiac motion. Imaging the brain one can use peripheral tracking as the distance between heart and a fingertip is similar to the distance between heart and brain.

Noise from respiratory and cardiac cycles may be removed by regression using the recorded data in a tape-and-filter strategy (7, 51, 54). An alternative strategy is noise removal as estimated from the data itself (6, 55, 56).

### Head Motion

Head motion can be mitigated through careful instruction and comfortable fixating strategies as cushions and straps. Several tracking systems to monitor head motion have been developed and are described below. The main challenge is real-time integration of motion-tracking data and image acquisition.

#### The Case for Prospective versus Retrospective Head Motion Correction

Retrospective and intra-image methods for head motion registration perform image acquisition and head position registration in the same data set. Attempts at motion correction within the data cannot correct for through-plane motion (57), and also cannot account for movements in previous scans with effects on field inhomogeneity and spin history (32). The method may introduce blurring artifacts through interpolation.

The solution is image acquisition with simultaneous prospective motion correction (57–60). Thesen and colleagues (58) developed Prospective Acquisition CorrEction (PACE) that acquired images for a volume while monitoring the head position and realigned the 3D grid to the head position before scanning the next volume in a stepwise process with high precision. The main disadvantage is that movements are not corrected until they are detected in the image, so rigid body transformation is still necessary.

#### Scanner-External Motion Tracking Strategies

The most common strategy for prospective, slice-by-slice head movement registration is to optically monitor a marker attached to the patient’s forehead with one or more video cameras and synchronize data continuously between scanner-external the camera space (where the head is) and the magnet space (where BOLD signals are recorded) (59–61). This requires are extra hardware (camera and marker setup), line of sight between camera and head marker, extra software for position registration and regular synchronization with the BOLD signal. In addition, the initial setup requires time [ca. 30 min (60)] and may be a constraint to the patient flow. If the system requires calibration for individual patients this prolongs the in-bore patient time and worsens motion artifacts akin to prolonged scan time. On the pro side, it is universally applicable to all scanner types and relatively cheap.

## Remaining Challenges

Power and colleagues (5) have shown that movement artifacts imitating functional connectivity correlations between brain regions persist even after on-line scanner motion corrections as proposed by Thesen and colleagues (58). Here, motion-induced artifacts occurred with movements in the order of a few tenths of a millimeter or less. They propose a skip-and-redo strategy of motion tracking and removal of acquired volumes with tracked movement artifacts over a chosen threshold. tSNR may provide a quality measure of functional connectivity data (3). Further information on postprocessing strategies for noise removal may be found in Ref. (3, 4, 6).

At present, scanner internal and external motion correction solutions exist, and their main application is to find the areas with excessive motion during the scan, so these data can be discarded and reacquired. The remaining problem is to integrate data continuously from a motion correction setup during image acquisition.

## Conclusion

In summary, movement artifacts are a problem in applications with low SNR, and they are exacerbated at high resolution and long acquisition times. Basic important strategies for motion reduction are comfortable patient positioning, instruction, and reminding of the importance to keep still during the scan. Fast imaging techniques are essential for short acquisition times. Common preprocessing techniques include realignment and six parameter rigid body transformation, and measures to detect motion, e.g., DVARS (percent mean signal change). Regression of physiological noise from cardiac and respiratory motion is recommended employing a nuisance modeling strategy, alternatively, if former is futile, as estimated from the data itself. External motion tracking yields best control of motion artifacts that may compromise data but requires extra equipment and setup. Its main challenge is real-time data integration. Prospective tracking of cardiac and respiratory cycles and head motion provide possibilities for motion correction. Head motion artifacts are ideally handled by correction using tracked parameters, or combined with a skip-and-redo strategy for movements over a chosen threshold.

## Author Contributions

The idea was conceived by IH, HC, and AC. The first draft was written by IH with input from KM, JN, and AO. All authors have contributed to manuscript writing and have read and approved the final manuscript, and have no conflicts of interest.

## Conflict of Interest Statement

The authors declare that the research was conducted in the absence of any commercial or financial relationships that could be construed as a potential conflict of interest.
